# Association of Angiotensin I Converting Enzyme Insertion/287 bp Deletion Polymorphisms and Proliferative Prostatic Diseases among Lebanese Men

**DOI:** 10.1155/2020/5959134

**Published:** 2020-02-07

**Authors:** Asmahan A. El Ezzi, Jordan M. Clawson, Mohammed A. El-Saidi, Wissam R. Zaidan, Abigail Kovash, Jeremy Orellana, AnnaKarina Thornock, Ruhul H. Kuddus

**Affiliations:** ^1^Radioimmunoassay Laboratory, Lebanese Atomic Energy Commission, Beirut, Lebanon; ^2^Department of Chemistry and Biochemistry, Lebanese University, Hadath, Lebanon; ^3^Department of Biology, Utah Valley University, Orem, UT, USA; ^4^Department of Strategic Management and Operations, Utah Valley University, Orem, UT, USA

## Abstract

**Background:**

Angiotensin I converting enzyme (ACE) insertion (I) and 287 bp Alu repeat DNA fragment deletion (D) polymorphisms have been indicated in various cancers. Here, we investigated I/D polymorphisms in prostate cancer (PCa) and benign prostate hyperplasia (BPH) among Lebanese men.

**Methods:**

Blood DNA extracted from 69 control subjects, 69 subjects with clinically confirmed PCa, and 69 subjects with clinical BPH, all the subjects were aged 50 years or older, was subjected to the polymerase chain reaction. The PCR products were resolved in polyacrylamide gels to determine II, ID, and DD genotypes. The odds ratios (OR), 95% confidence intervals (CI), and *p* values of the allele frequencies and genotype ratios were calculated for establishing possible association of the alleles and/or genotypes and PCa and/or BPH.

**Results:**

The proportions of II, ID, and DD genotypes were significantly different from Hardy–Weinberg equilibrium for BPH and PCa groups (but not the control group), mostly due to overabundance of the ID genotypes. There was no significant difference in the I and D allele frequencies between the control groups and the affected groups. The ratio of (DD + ID)/II is significantly lower among the control group compared to the BPH group (RR = 8.92, *p* values of the allele frequencies and genotype ratios were calculated for establishing possible association of the alleles and/or genotypes and PCa and/or BPH. *p* values of the allele frequencies and genotype ratios were calculated for establishing possible association of the alleles and/or genotypes and PCa and/or BPH.

**Conclusions:**

Our data indicate that the D allele of the I/D polymorphisms of the ACE gene is associated with increased risk of BPH, and the ID genotype is a risk factor for both BPH and PCa among Lebanese males.

## 1. Introduction

Prostate cancer (PCa) is the second most commonly diagnosed cancer among men worldwide and is the second leading cause of cancer death for men. In 2019, 174,650 new cases of PCa was diagnosed in the United States and the disease claimed the lives of 31,620 men in the United States that year [1]. Benign prostatic hyperplasia (BPH) is a noncancerous enlargement of the prostate gland that can occur as men age. In the United States, BPH affects nearly 70% of men between the ages of 60 and 69 and over 80% of men 70 years and older [2]. Both diseases appear to run in families and carry many of the same risk factors, including genetic and lifestyle factors [3]. Identifying genetic variations associated with prostatic diseases common in PCa and BPH may prove helpful in early diagnosis and treatment of PCa, given that PCa is a deadly disease and often difficult to diagnose early, whereas BPH is a relatively benign disease, but it displays some diagnostic characteristics at the early phases of the disease.

The human angiotensin I converting enzyme (ACE) gene encodes an enzyme that catalyzes the processing of angiotensin I to angiotensin II and angiotensin (1–7) to angiotensin (1–5) [4]. Angiotensin II is further processed by another enzyme, angiotensin converting enzyme 2 (ACE2) to angiotensin (1–7) [5]. Angiotensin II is a potent vasopressor and aldosterone-stimulating peptide involved in blood pressure control and fluid-electrolyte balance [4]. Angiotensin (1–7) is a potent vasoconstrictor and an effector component of angiotensin II, while angiotensin (1–5) stimulates secretion of atrial natriuretic peptide (ANP) [5, 6]. Overall, ACE plays a key role in the renin-angiotensin system by regulating fluid volume and controlling blood pressure. The ACE gene is located on chromosome 17 (17q23.3), and it has a 287 bp Alu repeat element insertion/deletion (I/D) polymorphism in intron 16. The primary transcript of the gene is differentially spliced, and different versions of the mature mRNAs are translated to synthesize various isoforms of the enzyme; of them, one isoform is adequately expressed in the testis [4].

Given that ACE plays a key role in the renin-angiotensin system that regulates fluid volume and controls blood pressure, it is highly likely that I/D polymorphism of the ACE gene could have diverse physiological consequences. Indeed, the I and D alleles of the ACE gene have been associated with an increased risk for many diseases [7–12]. Uemura et al. [10] observed a statistically significant association of the D allele and high systolic and diastolic blood pressure in the Japanese population and obesity, hyperlipidemia, hypertension, and diabetes mellitus more prevalent among the ID and DD genotypes, compared to the II genotype. In another study involving a Brazilian population, no direct association of I/D polymorphism of the ACE gene and G8790A polymorphism of the ACE2 gene and systemic arterial hypertension was observed although significant association of the combination of the DD genotype and GG genotype with systemic arterial hypertension was observed [11]. A study in southeast Turkey indicated that women with DD or ID genotypes had a 72% increased risk of idiopathic recurrent pregnancy loss [12], while another study in northern Iran found the DD genotype to be more prevalent among women who have experienced recurrent pregnancy loss [13]. Additionally, the I/D polymorphism has been found to be associated with Alzheimer's disease; however, both the I allele [14, 15] and the D allele [16] have been found to be associated with an increased risk of developing the disease. Other studies have found the I/D polymorphism to be associated with various cancers. For example, one study observed an association of the D allele with a 3-fold increased risk for glioma in an Algerian population [17], while a Polish study found the DD genotype to be associated with a 2-fold increased risk for multiple myeloma [18]. The I/D polymorphisms of the ACE gene have also been implicated in prostate cancer and breast cancer (reviewed in [19]). In the present study, we examined the association of the I/D polymorphism in the ACE gene and the risk of PCa and BPH among Lebanese men.

## 2. Materials and Methods

### 2.1. Subjects

All the subjects involved in this study were volunteer participants of prostate disorder screening campaigns organized by Prof. El Ezzi in collaboration with several hospitals/medical centers in Lebanon. An informed consent form to participate in the prostate-specific antigen (PSA) screening and associated investigative activities, including blood donation, extraction of DNA from blood cells, usage of DNA for genetic analysis, and use of the genetic data for research and publishing, was obtained from each subject in accordance with the ethical standards of the 1975 Declaration of Helsinki. The procedures and guidelines of the Institutional Review Board (IRB) of Utah Valley University were also followed (IRB approval #00614). Each participant was evaluated for prostate health by measuring the serum total PSA (PSA-T) level followed by a digital rectal examination (DRE) if necessary. A PSA assay kit obtained from Immunotech (Marseille, France) was used to quantify the PSA-T level. For the subjects with a PSA-T level in the gray zone (i.e., between 4 and 10 ng/ml), a free PSA test (PSA-F) was conducted and the PSA F/T ratio was determined to help differentiate between BPH and PCa. In addition, the International Prostate Symptom Score (IPSS) value was determined, and if necessary, a transrectal ultrasonography was conducted for appropriate cases. The subjects were considered control subjects if they had a normal level of PSA for two consecutive years, had a normal IPSS score, and a normal DRE at the time of sampling. The number of subjects with confirmed PCa was 69. Accordingly, 69 subjects with BPH and 69 control subjects were considered for the present study, yielding a total of 207 participants.

### 2.2. Molecular Methods

DNA was extracted from freshly collected whole blood samples using QiaAmp DNA Blood Mini Kit (Qiagen, Milan, Italy). DNA was quantified, and quality of DNA was assayed using a UV spectrometer. A fragment of the ACE gene flanking the I/D polymorphism was amplified by polymerase chain reaction (PCR) using the primers 5′-CTGGAGACCACTCCCATCCTTTCT-3′ and 5′-GATGTGGCCATCTTCGTCAGAT-3′ [9]. The PCR mixture (15 *μ*l) contained 1x reaction buffer containing 0.75 unit of Taq DNA polymerase (Qiagen, Germantown MD), 10 picomoles of the two primers, and 10 ng of template DNA. The negative control PCR conducted simultaneously lacked any template DNA. The reactions were set in a UV-decontaminated Class II A2 biosafety cabinet (equipped with high efficiency particulate air filter) using a dedicated set of pipettes to avoid cross contamination. A GeneAmp 2700 thermocycler (Applied Biosystems, Carlsbad, CA) was used for PCR amplification. The thermocycler was programed as follows: 94°C for 5 minutes (one cycle), 94°C for 45 sec, 58°C for 45 sec, and 72°C for 45 sec (35 cycles); 72°C for 5 minutes (one cycle); and soak at 4°C. Amplified DNA was resolved in 6% polyacrylamide gels for about 90 minutes at 80 volts using 0.5x Tris-Borate-EDTA buffer as the electrolyte. The gels were stained for 20 minutes in ethidium bromide (0.5 *μ*g/ml in 0.5x Tris-borate-EDTA buffer). The DNA bands were visualized on a UV transilluminator and then documented using a digital camera. The insertion (I) allele generated a 490 bp DNA fragment, while the deletion allele (D) generated a 290 bp DNA fragment with the primer set used.

### 2.3. Statistical Analysis

The frequencies of the ACE gene I/D genotypes (II, ID, and DD) of the three samples in conjunction with Hardy–Weinberg (H-W) equilibrium were used to perform the χ^2^ goodness-of-fit. The null hypothesis that the genotypes of the three populations are in H-W equilibrium were rejected at the 0.05 level of significance if the calculated *χ*^2^ test statistic produced a *p* value smaller than 0.05. Given the possibility of three genotypes (II, ID, and DD) and two alleles (I and D), the number of degrees of freedom was one. Association of the polymorphic genotypes with PCa and BPH was evaluated by calculating the odds ratio (OR) and the 95% confidence interval (CI). The relative risk (RR) for having a genotype in developing PCa or BPH along with the associated 95% CI was also calculated. For all statistical tests conducted, a *p* value less than 0.05 was considered significant. The OR, RR, 95% CI, *p* value, and the difference of the mean age of the control group and the affected groups were calculated using MedCalc Statistical Software for Biomedical Research (MedCalc Software, Acacialaan 22, B-8400 Ostend, Belgium).

## 3. Results

This study included 69 cases of PCa (mean age 66.4 ± 8.5 years), 69 subjects with confirmed BPH (mean age 69.1 ± 8.4 years), and 69 control subjects with no known prostate pathology (mean age 55.8 ± 11.0 years). The mean age of the control group is significantly different from that of PCa and BPH (*p* value <0.001). However, the mean age of the PCa and BPH groups is not significantly different (*p* value = 0.062).

### 3.1. Distribution of the Genotypes and Allele Frequencies

The distribution of homozygous insertion (II), homozygous deletion (DD), and the heterozygous (ID) genotypes in the three samples is shown in Table 1. The distribution of the genotypes of the control group is in H-W equilibrium. However, the ID genotype is overrepresented in both the PCa and BPH groups, making the distribution of the three genotypes significantly different from H-W equilibrium for the PCa group (*χ*^2^ = 5.61, *p* value = 0.018) and the BPH group (*χ*^2^ = 17.62, *p* value <0.001).

The frequency and the proportion of the I and D alleles among the control subjects and the PCa and BPH groups are shown in Table 2. There is no significant difference in the distribution of the I and D alleles between the control group and PCa group (OR = 1.24, 95% CI = 0.76–2.01, *p* value = 0.387), between the control group and BPH group (OR = 1.0, 95% CI = 0.16–1.63, *p* value = 1.00), or between the control group and the combined affected groups (OR = 1.11, 95% CI = 0.073–1.70, *p* value = 0.616).

### 3.2. Genotype Frequencies and Genotypic Ratios

The frequency of II, DD, and ID genotypes for the control group and the PCa and BPH groups along with the OR, RR of PCa or BPH, 95% CI, and the *p* values are shown in Table 3. There is no significant difference in the ratios DD/II, DD/ID, (DD + ID)/II, (ID + II)/DD, or ID/(II + DD) for the control subjects and the subjects with PCa (Table 3). However, the ratio of (DD + ID)/II of the control group and subjects with BPH are significantly different (OR = 8.92, 95% CI 1.08–73.37, *p* value = 0.042 and the corresponding RR = 1.11, 95% CI 1.02–1.22, *p* value = 0.018). The DD to II ratio for the control group is also significantly lower compared to subjects with BPH (OR = 5.93, 95% CI 0.682–51.27, *p* value = 0.106; the corresponding RR = 1.23, 95% CI 1.00–1.51, *p* value = 0.043), indicating that the D allele is associated with increased risk of BPH. In addition, the ID/(II + DD) ratio is significantly lower among the control subjects compared to the subjects with BPH (RR = 2.35, 95% CI 1.17–4.72, *p* value = 0.016 and the corresponding RR = 1.41, 95% CI 1.06–1.88, *p* value = 0.018), indicating that the ID genotype is a risk factor for BPH. The ID/(II + DD) ratio is also significantly lower among the control subjects compared to the affected subjects (OR = 1.99, 95% CI 1.10–3.59, *p* value = 0.021; and the corresponding RR = 1.34, 95% CI 1.02–1.75, *p* value = 0.032), again indicating that the ID genotype is potentially a risk factor for PCa and BPH subjects.

## 4. Discussion

Lebanon is a small country with a population of 6.1 million. In 2018, a total of 8809 new cases of cancers were diagnosed among the male population of the country. Prostate cancer was the most diagnosed cancer (17.1% of all cancer cases) among males in that year, and overall, prostate cancer was the fourth most diagnosed cancer (behind breast, bladder, and lung cancers) in the country [20]. The age-standardized incidence rate of prostate cancer changed from 27.6 cases/100,000 in 2003 [21] to 39.2 cases/100,000 by 2008 indicating a 7.6% increase [22]. We were unable to find any published data on BPH incidence in Lebanon, but the incidence is expected to be high, given that BPH is an age-associated disease [2] and the relatively high life expectancy (78 years) of Lebanese men [23]. In this study, we examined the association of the I/D polymorphism with PCa and BPH among Lebanese men.

Our preliminary analyses showed no association of I/D polymorphism and PCa or BPH among Lebanese men [24]. However, a more critical analysis involving confirmed cases of PCa and BPH presented here indicated a positive association of the D allele with BPH and confirmed a lack of association between I/D polymorphism and the risk of PCa. However, our data indicate that the ID genotype is a risk factor for both PCa and BPH. A previous study [25] indicated that the I allele is protective, while the D allele is associated with PCA, and the DD genotype is related to aggressive stage of PCa in the Han population of China. Another study in Iran indicated that subjects with II genotype have a higher PSA level than subjects with DD genotype [26]. The same study also indicated that II genotype is associated with BPH and the D allele is associated with PCa risk, but the associations were not statistically significant [26]. A study in the Mexican population found the D allele to be associated with increased risk of both PCa and BPH [27]. A study in Turkey also found the D allele associated with increased risk with PCa, and the DD genotype was found to contain a higher level of PSA-T [9]. A metanalysis involving 35 published studies indicated lack of association of I/D alleles or genotypes and risk of PCa or any other cancers although the I allele and II genotype were found to be associated with increased cancer risk among Caucasians [8]. A more recent metanalysis analyzing multiple study found no association of I/D polymorphism and PCa risk overall and among the Caucasian males in particular although the study found the D allele and the DD genotype associated with increased risk of PCa among the Asian and Latino males [28]. Similar inconsistencies were observed when the association of the I/D polymorphism and other cancers such as breast cancer [29, 30], lung cancer [31, 32], and colorectal cancer [33, 34] was investigated.

The above discussion indicates that although the D allele appears to be a risk factor for increased cancer risk [9, 25–28], the I/D polymorphism is generally not a risk factor for BPH, PCa, or other cancers, and one of the alleles or genotypes can be associated with increased risk of cancer in certain ethnic groups. Many different factors including a possible polygenic basis for the diseases, variability in penetrance, and the environmental conditions different ethnic and racial groups encounter [35] may contribute to such variable clinical outcomes. If multiple genes were involved in PCa, BPH, or any cancers and if some of the genes were polymorphic, a subject might have some protective and some risk-bearing alleles of the genes [36], adding additional layers of influences on the phenotypic outcomes. A system biology approach such as the genome-wide association studies (GWAS) is currently being applied to address the issue [37, 38]. However, identification of important genetic polymorphisms relevant to disease risks of different ethnic groups would remain useful given that genetic polymorphisms are extremely common.

## 5. Conclusion

The present data indicates that the D allele of the I/D polymorphism is associated with increased risk of BPH and the ID genotype is associated with increased risk of BPH and PCa among Lebanese males.

### 5.1. Limitations

The small population of Lebanon and the smaller number of males volunteered to participate in the study contributed to some limitations of the present study. The overall sample sizes of the control and the PCa and BPH groups are relatively small, and although all participants of the study were 50 or older, the difference in the mean age of the control group and the affected groups is statistically significant.

## Figures and Tables

**Table 1:** Allelic distribution of ACE gene among the control subjects (*n* = 69) and the subjects with BPH (*n* = 69) or PCa (*n* = 69).

Groups	Observed frequency (II : ID : DD)	Expected frequency (II : ID : DD)	*χ* ^2^ statistic	*p* value
Control	8 : 34 : 27	9.06 : 31.88 : 28.06	0.304	0.581
PCa	7 : 43 : 19	11.77 : 33.46 : 23.77	5.61	0.018^*∗*^
BPH	1 : 48 : 20	9.06 : 31.88 : 28.06	17.62	<0.001^*∗*^

^*∗*^Significant at the 0.05 level of significance.

**Table 2:** The frequencies (and proportions) of the I and D alleles of the ACE gene among the controls and the subjects with PCa or BPH.

Alleles	Control	Affected	OR	95% CI	*p* values
*A. Control group versus PCa group*					
I	50 (0.36)	57 (0.41)	1		
D	88 (0.64)	81 (0.59)	1.24	0.76–2.01	0.387

*B. Control group versus BPH group*					
I	50 (0.36)	50 (0.36)	1		
D	88 (0.64)	88 (0.64)	1.00	0.61–1.63	1.00

*C. Control group versus the combined PCa and BPH groups*					
I	50 (0.36)	107 (0.39)	1		
D	88 (0.64)	169 (0.61)	1.11	0.73–1.70	0.616

**Table 3:** The frequencies of the genotypes II, ID, and DD of the control and the subjects with PCa or BPH.

Allele	Control	Affected	OR	95% CI	*p* value	RR	95% CI	*p* value
*A. Control group versus PCa group*								
DD/II	27/8	19/7	0.80	0.25–2.60	0.716	0.95	0.71–1.27	0.719
DD/ID	27/34	19/43	0.56	0.27–1.17	0.120	0.69	0.43–1.11	0.124
(DD + ID)/II	61/8	62/7	1.16	0.40–3.40	0.785	1.02	0.90–1.14	0.785
(II + ID)/DD	42/27	50/19	1.69	0.83–3.46	0.150	1.19	0.94–1.51	0.152
ID/(DD + II)	34/35	43/26	1.70	0.86–3.35	0.124	1.26	0.94–1.71	0.127

*B. Control group versus BPH group*								
DD/II	27/8	20/1	5.93	0.68–51.27	0.106	1.23	1.00–1.51	0.043^*∗*^
DD/ID	27/34	20/48	0.52	0.25–1.08	0.082	0.66	0.42–1.06	0.084
(DD + ID)/II	61/8	68/1	8.92	1.08–73.37	0.042^*∗*^	1.11	1.02–1.22	0.018^*∗*^
(II + ID)/DD	42/27	49/20	1.58	0.77–3.20	0.210	1.17	0.92–1.49	0.212
ID/(DD + II)	34/35	48/21	2.35	1.17–4.72	0.016^*∗*^	1.41	1.06–1.88	0.018^*∗*^

*C. Control group versus the combined PCa and BPH (affected) groups*								
DD/II	27/8	39/8	1.44	0.48–4.32	0.512	1.08	0.86–1.34	0.520
DD/ID	27/34	39/91	0.54	0.29–1.01	0.055	0.68	0.46–0.99	0.048^*∗*^
(DD + ID)/II	61/8	130/8	2.13	0.76–5.95	0.148	1.07	0.97–1.17	0.190
(II + ID)/DD	42/27	99/39	1.63	0.88–3.00	0.115	1.18	0.95–1.46	0.136
ID/(DD + II)	34/35	91/47	1.99	1.10–3.59	0.021^*∗*^	1.34	1.02–1.75	0.032^*∗*^

^*∗*^Significant at the 0.05 level of significance.

## Data Availability

The detailed genotyping data of the patients are available from the corresponding author and only to be made available to researchers who meet the criteria for access.
